# Ghrelin Expression in Atherosclerotic Plaques and Perivascular Adipose Tissue: Implications for Vascular Inflammation in Peripheral Artery Disease

**DOI:** 10.3390/jcm13133737

**Published:** 2024-06-26

**Authors:** Sorin Nicolae Peiu, Diana Gabriela Iosep, Mihai Danciu, Veronica Scripcaru, Victor Ianole, Veronica Mocanu

**Affiliations:** 1 Vascular Surgery Department, “Grigore T. Popa” University of Medicine and Pharmacy, 700115 Iasi, Romania; sorin-nicolae.peiu@umfiasi.ro; 2 Morpho-Functional Sciences II (Physiopathology) Department, “Grigore T. Popa” University of Medicine and Pharmacy, 700115 Iasi, Romania; veronica.mocanu@umfiasi.ro; 3 Pathology Department, “Sf. Spiridon” Emergency Clinical Hospital, 700111 Iasi, Romania; 4 Morpho-Functional Department—Morphopathology, “Grigore T. Popa” University of Medicine and Pharmacy, 700115 Iasi, Romania; veronica.scripcaru@umfiasi.ro (V.S.); victor.ianole@umfiasi.ro (V.I.)

**Keywords:** peripheral artery disease, atherosclerosis, ghrelin, vascular inflammation, plaque morphology, immunohistochemistry, metabolic syndrome

## Abstract

Atherosclerosis, a leading cause of peripheral artery disease (PAD), is driven by lipid accumulation and chronic inflammation within arterial walls. **Objectives**: This study investigates the expression of ghrelin, an anti-inflammatory peptide hormone, in plaque morphology and inflammation in patients with PAD, highlighting its potential role in age-related vascular diseases and metabolic syndrome. **Methods**: The analysis specifically focused on the immunohistochemical expression of ghrelin in atherosclerotic plaques and perivascular adipose tissue (PVAT) from 28 PAD patients. Detailed immunohistochemical staining was performed to identify ghrelin within these tissues, comparing its presence in various plaque types and assessing its association with markers of inflammation and macrophage polarization. **Results**: Significant results showed a higher prevalence of calcification in fibro-lipid plaques (63.1%) compared to fibrous plaques, with a notable difference in inflammatory infiltration between the two plaque types (*p* = 0.027). Complicated plaques exhibited increased ghrelin expression, suggesting a modulatory effect on inflammatory processes, although this did not reach statistical significance. The correlation between ghrelin levels and macrophage presence, especially the pro-inflammatory M1 phenotype, indicates ghrelin’s involvement in the inflammatory dynamics of atherosclerosis. **Conclusions**: The findings propose that ghrelin may influence plaque stability and vascular inflammation, pointing to its therapeutic potential in managing atherosclerosis. The study underlines the necessity for further research to clarify ghrelin’s impact on vascular health, particularly in the context of metabolic syndrome and age-related vascular alterations.

## 1. Introduction

Atherosclerosis, a chronic inflammatory disease predominantly affecting large and medium-sized arteries, is primarily caused by lipid metabolism dysregulation and maladaptive inflammatory responses [1]. This condition is the fundamental pathological basis of peripheral artery disease (PAD), which includes atherosclerosis of the abdominal aorta, iliac, and lower-extremity arteries.

Classically, the pathophysiology of atherosclerosis is characterized by internal factors in the vessel wall, such as endothelial dysfunction, lipid deposition, inflammation, and smooth muscle cell proliferation, ultimately leading to plaque formation [2]. Multiple cell types are involved in atherosclerosis; however, macrophages are critical in the progression and complications associated with advanced stages of the disease [3,4].

Within the inflammatory microenvironment of the lesion, macrophages polarize toward two major phenotypes that participate in the development of atherosclerosis through different pathways. Pro-inflammatory M1 macrophages are the main inflammatory cell population in lipid cores [5,6]; they engulf oxidized low-density lipoprotein (LDL) cholesterol and transform into foam cells, are responsible for the formation of a necrotic core, and promote disease progression and plaque rupture. Anti-inflammatory M2 macrophages, on the other hand, phagocytize lipid particles and apoptotic cells, and stimulate plaque regression [7,8,9]. The dynamic evolution and stability of atherosclerotic plaques rely on both the quantity of infiltrated macrophages and their polarization state [7].

New findings suggest that perivascular adipose tissue (PVAT) may play a role in worsening atherosclerosis through an “outside-in” mechanism [10,11]. Physiologically, PVAT is a modulator of vascular and metabolic homeostasis that exerts protective vasodilatory, anti-inflammatory, and antioxidative effects [12,13]. Under pathological conditions such as obesity, metabolic syndrome, and aging, PVAT becomes dysfunctional and, in turn, exacerbates endothelial dysfunction, increases local macrophage infiltration, and thus possibly contributes to the pathogenesis of atherosclerotic lesions [14,15,16].

Ghrelin, an orexigenic peptide hormone discovered in 1999, has attracted research interest as a potential target in treating obesity and cardiovascular diseases [17,18]. Ghrelin has been found to enhance free fatty acid oxidation and reduce glucose oxidation in heart failure by partially correcting metabolic alterations in heart failure. By increasing the levels of circulating des-acyl or acyl ghrelin, a partial reversal of energy substrate metabolic alterations in failing hearts was achieved by lowering glucose oxidation while stimulating free fatty acid oxidation [19]. In vitro studies have shown beneficial effects of ghrelin administration in ischemic limbs due to activation of proangiogenic microRNAs [20]. The role of endogenous ghrelin in vascular homeostasis and therapeutic angiogenesis has been investigated by experiments in ghrelin knockout mice with diabetes-induced PAD. Treatment with acylated ghrelin (AG) and des-acyl ghrelin (DAG) resulted in decreased fibrosis in hindlimb ischemia-induced lesions, but the antifibrotic effects were higher in AG compared to DAG [21]. PAD is also associated with an increase in homocysteine levels, which act not only on coronary arteries but also on the peripheral vasculature. Ghrelin ameliorates homocysteine-induced vascular injury, and this could also be studied in PAD [22]. Ghrelin was able to limit the activation of endoplasmic reticulum stress, which is responsible for the deregulation of lipid metabolism that characterizes all phases of atherosclerotic lesion and plaque formation [23].

Predominantly found in gastric oxyntic glands and in vascular and cardiac tissues [24,25,26], ghrelin has multifaceted functions, including appetite stimulation, white adipose tissue expansion, and exerting vasodilatory and anti-inflammatory effects on arteries [27,28]. Although earlier studies suggested that the vasodilatory effects of ghrelin may be independent of GH/IGF-I/nitric oxide (NO) and related to the autonomic nervous system, as ghrelin stimulates gastric acid secretion by activating the vagal system [29], it was shown that the vasodilatory effect of ghrelin is associated with the activation of endothelial nitric oxide synthase (eNOS), the main source of vascular NO [30]. NO leads to a reduction in intracellular Ca^2+^ and vasodilation. Ghrelin receptor GHS-R1a induces IP3 production, which causes phospholipase C-protein kinase C (PLC/PKC)-dependent Ca^2+^ mobilization by L-type voltage-gated calcium channels [31]. A constrictor effect of ghrelin on the coronary vasculature dependent on Ca^2+^ and PKC activation was found in the rat coronary vasculature [32].

Multiple studies have associated ghrelin with the inhibition of atherosclerotic plaque formation and promotion of plaque stability [33,34,35,36], with observations of lower circulating ghrelin levels in obese patients with metabolic syndrome [37]. However, the interplay between ghrelin and macrophages in atherosclerotic plaques and adjacent fat tissue is not well defined.

Given the link between obesity, insulin resistance (metabolic syndrome, type 2 diabetes mellitus), and a spectrum of cardiovascular diseases, including PAD, the impact of ghrelin on vascular dysfunction in patients with these comorbidities warrants investigation [38]. This study examines atherosclerotic plaque morphology, inflammation in the arterial wall and PVAT, and ghrelin immunohistochemical expression in plaques and perivascular tissues, aiming to elucidate ghrelin’s role in vascular inflammation in patients with age-related vascular diseases and metabolic syndrome.

## 2. Materials and Methods

### 2.1. Study Group

The study cohort included 28 patients admitted to the Vascular Surgery Clinic and diagnosed with peripheral arterial disease. Inclusion criteria were based on clinical signs and angiographic data supporting the diagnosis of peripheral arterial disease with critical limb ischemia (intermittent claudication, rest pain, ischemic ulceration, or gangrene) with indications for an endovascular approach. Exclusion criteria included patients associated with oncological diseases to avoid paraneoplastic syndromes. A detailed description of the study group is provided in Table 1.

### 2.2. Tissue Collection

Surgical interventions involved distal femoropopliteal bypass with reversed saphenous vein, femoropopliteal bypass with reinforced PTFE synthetic graft, aorta-bifemoral bypass with silver-coated Dacron synthetic graft, and lower limb amputations at the level of the calf and thigh. Tissue samples were obtained from the superficial femoral artery, popliteal artery, anterior tibial artery, and external iliac artery.

Arterial and perivascular adipose tissues were harvested from patients with peripheral arterial disease. For optimal clinical relevance, areas with advanced atherosclerosis were selected based on angiographic images obtained during atherectomy. The control group included 15 samples of normal muscular arteries and perivascular adipose tissue of lower limbs, surgically harvested from young patients who were victims of traffic accidents.

### 2.3. Histological Processing of Tissues

The tissue samples were macroscopically examined and described, varying in size from 0.5 cm to 18 cm in length. These tissues were routinely processed by fixation in 10% buffered formalin (for 12–20 h depending on the sample’s size), paraffin embedding, sectioned at 4 µm, and stained with hematoxylin-eosin for optical microscope analysis. After fixation, tissue fragments with calcified atherosclerotic plaques were decalcified for 2–4 h (Biodec R, Bio-Optica, Milan, Italy). Pathological assessment was independently performed by three experienced pathologists. The evaluation focused on plaque type, complications, and semi-quantitative assessment of the inflammatory infiltrate.

### 2.4. Immunohistochemical Study

In this study, we investigated the immunoexpression of the following markers: CD68—general macrophage marker, CD80—for M1 macrophages, and ghrelin. Their expression was assessed in atherosclerotic plaques and in the perivascular adipose tissue.

Immunohistochemical analyses employed monoclonal antibodies, specifically anti-CD68 (rabbit anti-human, dilution 1:100, clone 514H12, Novocastra, Newcastle, UK), anti-CD80 (rabbit anti-human, dilution 1:100, clone EPR1157, Abcam, Cambridge, UK), and anti-ghrelin (rabbit anti-human, dilution 1:50, clone EPR20502, Abcam, Cambridge, UK). The pretreatment was performed using a specific epitope retrieval solution (pH 9) at 96 °C for 25 min. For the detection of immunoreactive signals, the UltraVision LP Detection System and 3,3′-Diaminobenzidine (DAB) chromogen, both sourced from ThermoFisher Scientific, Fremont, CA, USA, were utilized. To ascertain the specificity of immunoreactivity, primary antibodies were omitted and replaced with non-immunized serum at equivalent dilutions (negative control) for each sample. Furthermore, to ensure reproducibility and accuracy, each sample underwent two replicates for immunohistochemical testing.

For semi-quantitative evaluation of the markers CD68, CD80, and ghrelin, the following scoring system was used: 0 ≤ 1% positive cells, 1 = 1–29% of tissues showing positivity, 2 = 30–59%, 3 ≥ 60%.

The intensity of the ghrelin immunolabeling was scored as follows: 0 = negative, 1 = weak, 2 = moderate, 3 = strong.

For total ghrelin assessment, we determined a semi-quantitative score obtained from the sum of the intensity and proportion of positive cells. We determined the graphical distribution of the score in complicated plaques and in their perivascular tissue by determining the receiver operating characteristic (ROC) curve and calculating the area under the curve (AUC). Calculation of the ROC curve by determining the distribution of ghrelin score assessed in complicated atherosclerotic plaques recorded a very good value of AUC = 0.867, with a cut-off point of 1 and with a satisfactory value in perivascular tissue of AUC = 0.654, with a cut-off point of 1. Therefore, two groups were formed: 0 and 1 were assigned a low expression, and 2–6 were assigned a high expression.

### 2.5. Statistical Analysis

Data were statistically analyzed using IBM Statistical Package for the Social Sciences (SPSS) version 19. Relationships between complications and plaque type, and between immunohistochemical markers in the plaque and perivascular tissue (CD68, CD80, and ghrelin) and plaque type or complications, were determined through Fisher’s exact test. A *p*-value < 0.05 was considered statistically significant.

## 3. Results

### 3.1. Study Group Characteristics

The overall patient age ranged from 53 to 86 years old, with a mean age of 69 years old. The distribution of cases by age group recorded a maximum in the 60–69-year-old age group (53.6%). The correlation of patient age with plaque type did not show statistically significant values (*p* = 0.666 for plaque type and *p* = 0.207 for plaque complications).

Table 2 shows the distribution of patients by gender, with a higher incidence in males compared to females and a sex ratio of M/F = 3/1.

### 3.2. Pathological Evaluation

In the study group, morphological changes present in the arterial wall, the atherosclerotic plaque type, its complications, and inflammatory infiltration in the arterial wall and in its perivascular tissue were analyzed.

The most frequent type of atherosclerotic lesions was fibrous plaques (57.1%), followed by fibro-lipid plaques (42.9%).

**Figure 1:**
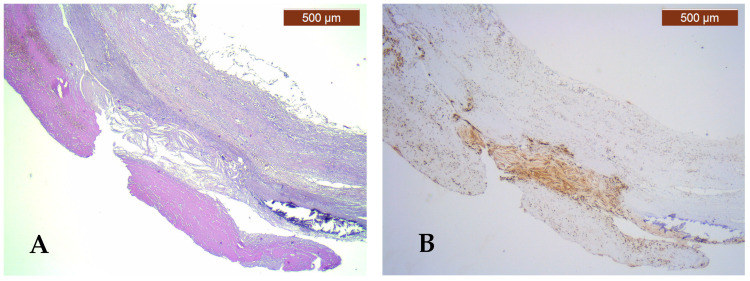

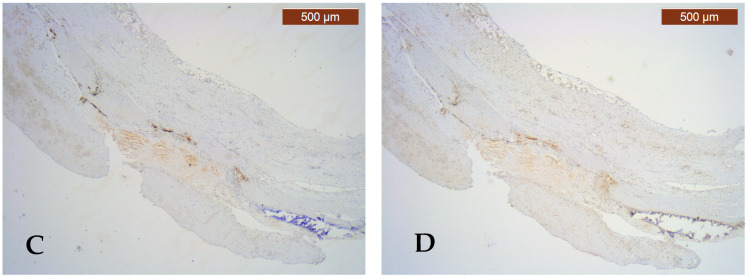
Pathological and immunohistochemical study of the atherosclerotic plaque and PVAT. (**A**) Fibro-lipid plaque complicated with calcification, ulceration, and thrombosis (HE, ×25); (**B**) strongly positive CD68 immunoexpression (score 3) of numerous macrophages in the complicated plaque and negative CD68 immunoexpression (score 0) for macrophages in PVAT (IHC, anti-CD68 antibody, ×25); (**C**) moderately positive CD80 immunoexpression (score 2) for M1 macrophages in the complicated plaque and negative CD80 immunoexpression (score 0) for M1 macrophages in PVAT (IHC, anti-CD80 antibody, ×25); (**D**) high expression of ghrelin (intensity score 2 and proportion score 3) in the complicated plaque and low ghrelin expression (intensity score 0 and proportion score 0) in PVAT (IHC, anti-ghrelin antibody, ×25).

Various complications affecting atheromatous plaques were identified, such as ulceration, thrombosis, calcification, and bone metaplasia (Figure 1A). The most common complication encountered in the arterial wall with atherosclerotic lesion in the studied group was calcification (67.9%). The distribution of calcification according to plaque type revealed a higher incidence of calcified fibro-lipid plaques (63.1%), with statistically significant differences (*p* = 0.003). Ulcerated plaques were identified in 35.7% of the cases, of which 70% were ulcerated fibro-lipid plaques and 30% were ulcerated fibrous plaques. Thrombosis was identified in 46.4% of cases, with a slightly higher incidence in fibro-lipid plaques (53.9%) compared to fibrous plaques (46.1%); these differences were not statistically significant (*p* = 0.445) (Table 3). The lowest incidence of complicated atherosclerotic lesions was bone metaplasia, being present in 10.7% of cases.

The presence of inflammatory infiltrate was assessed in atherosclerotic plaques and in perivascular fat tissue.

The distribution of inflammatory infiltrate according to plaque type revealed the presence of inflammatory infiltrate in 91.6% of fibro-lipid plaques and in 50% of fibrous plaques, with statistically significant differences (*p* = 0.027).

Inflammatory infiltrate was present in 82.6% of complicated plaques and being absent in uncomplicated plaques, with statistically significant differences (*p* = 0.002).

Quantification of inflammation in perivascular tissue did not show statistically significant variations either by plaque type (fibrous/fibro-lipidic) or by complication.

The distribution of inflammation type showed the presence of a lymphoplasmacytic inflammatory infiltrate in 87.5% of fibrous plaques and in 72.72% of fibro-lipidic plaques, with no statistically significance values.

Lymphoplasmacytic inflammatory infiltrate was identified in 78.94% of complicated plaques and was absent in uncomplicated plaques, with statistically significant differences (*p* = 0.003).

No significant difference was found in the distribution of inflammation type according to perivascular tissue (*p* = 0.148 in perivascular tissue adjacent to plaque type and *p* = 0.795 in perivascular tissue adjacent to complicated/uncomplicated plaques) (Table 4).

### 3.3. Immunohistochemical Study

The immunoexpression of CD68, CD80, and ghrelin was determined in atherosclerotic plaques and in their perivascular adipose tissue.

#### 3.3.1. CD68 Expression

In fibrous plaques, CD68 immunolabelling showed the presence of macrophages in different proportions in all fibro-lipid plaques, and only 50% of fibrous plaques, with statistically significant values (*p* = 0.017).

CD68 immunostaining was present in varying proportions in 82% of complicated atherosclerotic plaques (Figure 1B) and in only 20% of uncomplicated atherosclerotic plaques, but with no statistically significant values (*p* = 0.074).

In connective tissue adjacent to arteries, CD68 expression was positive in varying proportions in 58.3% in tissue adjacent to fibrous plaques and in 70% in tissue adjacent to arteries with fibro-lipid plaques, with no statistically significant values (*p* = 0.809).

CD68 expression was positive in 66.6% of both perivascular tissue adjacent to uncomplicated and complicated plaques, with no statistical significance (*p* = 0.367) (Table 5).

#### 3.3.2. CD80 Expression

Immunoexpression of the CD80 marker showed a higher presence of M1 macrophages in fibro-lipid plaques compared to fibrous plaques, with statistically significant differences (*p* = 0.007) (Figure 1C).

In complicated plaques, CD80 immunolabelling showed in 73.9% the presence of M1 macrophages in different proportions, with no statistical significance (*p* = 0.272).

Evaluation of CD80 immunoexpression of M1 macrophages in perivascular tissue in the vicinity of plaques revealed slightly higher immunoexpression adjacent to fibrous plaques (43%) compared to tissue around fibro-lipid plaques (30%) but without statistically significant differences (*p* = 0.594).

In perivascular connective tissue in the vicinity of complicated atherosclerotic plaques, CD80 immunolabelled M1 macrophages were present in 42.8% in different proportions, with no statistically significant variations (*p* = 1.000) (Table 5).

#### 3.3.3. Ghrelin Expression

Ghrelin immunolabelling was evaluated semi-quantitatively by counting positive cells, as well as the intensity of the expression.

Semi-quantitative ghrelin scores were assessed in 15 arteries with complicated plaques and revealed a low expression in 26.6% and a high expression in 73.4%, differences with no statistical significance (*p* = 0.313) (Figure 1D).

In perivascular tissue in the vicinity of complicated plaques, the ghrelin score had low expression in 63.6% and high expression in 36.4%, values with no statistical significance (*p* = 1.000) (Table 6).

## 4. Discussion

Ghrelin is a peptide hormone produced mainly in the stomach that has widespread tissue distribution and affects all body systems. Since its discovery in 1999, accumulating investigations have proven that ghrelin possesses anti-inflammatory effects and a variety of beneficial cardiovascular activities [39]. Different studies conducted among humans have demonstrated that ghrelin is associated with atherosclerotic plaque stability [40] by inhibiting vascular endothelial cell apoptosis, improving endothelial dysfunction, and reducing vascular inflammation [41,42]. These findings highlight ghrelin’s potential as an antiatherogenic agent in the treatment of several cardiovascular diseases, such as PAD.

We sought to identify the connection between endogenous ghrelin and vascular inflammation in atherosclerosis, from both an “outside-in” and “inside-out” perspective. Our research focused on analyzing the expression of ghrelin in atherosclerotic plaques and PVAT, in association with inflammatory macrophage content and phenotype, as well as histopathological characteristics of plaques in PAD patients presenting with critical limb ischemia and metabolic syndrome.

PAD is an increasingly prevalent condition defined by stenosis or occlusion of the arteries supplying the lower limbs. The end-stage form of the disease, described as chronic or critical limb ischemia (CLI), affects up to 10% of patients with PAD and is characterized by a high risk of major amputation and mortality. CLI is primarily driven by progressive atherosclerotic disease [43].

Atherosclerosis is a chronic inflammatory disease characterized by intimal plaque formation in large and medium-sized arteries. Classically, the atherosclerotic process is initiated by endothelium activation, followed by a cascade of events: leukocyte recruitment, lipid accumulation, and fibrous tissue formation (“inside-out” mechanism) [44]. Emerging evidence has brought to attention that PVAT, a specialized type of adipose tissue surrounding most blood vessels, also contributes to vascular inflammation in an “outside-in” manner [10]. Originally considered a support tissue, PVAT is now regarded as an endocrine organ that modulates vascular tone and vascular remodeling by secreting pro- and anti-inflammatory molecules [14]. PVAT is composed mainly of white adipocytes, and to a lesser extent brown adipocytes, macrophages, lymphocytes, and fibroblasts [45]. Under pathological conditions, such as metabolic disorder, chronic inflammation, and aging, PVAT undergoes phenotypic changes and becomes a source of inflammatory factors [11] that promote monocyte migration into the vessel wall and macrophage activation [46]. Since PVAT and the vessel wall are interconnected through a bidirectional signaling pathway, the development of atherosclerosis and plaque complications are now considered to be influenced by the combination of “inside-out” and “outside-in” mechanisms [47].

Atherosclerotic plaques are characterized by heterogeneous structures, and plaque composition is directly connected with the risk of plaque rupture [48]. The vulnerable plaque is characterized by a large necrotic core, thin fibrous cap, spotty calcification [49], and increased inflammatory cell infiltrate [50]. In the late stage of atherosclerosis, plaque instability eventually causes complications to arise, such as plaque rupture, bleeding, and thrombosis, clinically manifesting as CLI [51].

Although the pathology of atherosclerosis in the coronary and carotid arteries has been well studied, plaque characteristics in the lower extremities remain poorly understood [52]. Several histopathological studies focusing on patients with CLI showed that the main plaque type encountered in the femoral artery according to the American Heart Association (AHA) classification [53] was fibro-calcified plaque [54,55]. Lower-extremity artery plaques in CLI patients were reported to generally exhibit calcifications [54] to a more significant degree than carotid or coronary plaques [56,57]. Indeed, in our study, plaque morphology analysis showed greater association of fibro-lipid plaques with intimal calcifications as a possible feature of increased vulnerability. Previous studies of human coronary arteries illustrated that small calcium depositions increase the probability of atherosclerotic plaque rupture, in contrast to large areas of calcification at the intimal–medial site, which seems to prevent such a complication [58].

Macrophages are the most abundant type of immune cells in the atherosclerotic plaques, and they play a significant role in their development, progression, and rupture [59]. Both the number of infiltrated macrophages and the predominant phenotype dictate atherosclerotic plaque stability [4]. Specifically, classically activated M1 macrophages initiate and sustain inflammation [60], leading to fibrous cap thinning, foam cell formation, and necrotic core formation [61]. In symptomatic plaques, M1 macrophages were previously described as the abundant cell type in the developed lipid core [62].

Using immunohistochemistry, Shaikh et al. observed correlations between total macrophage count, M1 macrophage phenotype, and morphological parameters linked to plaque instability in carotid and femoral artery symptomatic atherosclerotic plaques. Additionally, the lipid core size and lymphocytic cellular infiltrate were more significantly correlated with M1 macrophages than the total macrophage count across all plaques studied [50]. Similarly, our study showed a more significant total macrophage count (CD68+) in fibro-lipid plaques than fibrous plaques, with a statistically greater association between M1 macrophages (CD68+, CD80+) and fibro-lipid plaques. This finding further supports the view that the proportion of the M1 macrophage subset within plaques is a better predictor of plaque composition and stability in comparison to total macrophages.

While we have not been able to note a connection between inflammatory macrophage content and phenotype in PVAT and atherosclerotic plaque morphology, previous studies also showed conflicting results. In an autopsy study, Farias-Itao et al. correlated histological components of unstable plaques (higher lipid content, decrease in fibrous cap thickness, and smooth muscle cell area) and plaque thrombosis with the presence of M1 macrophages in the periplaque PVAT in the coronary arteries [15]. In contrast, Fitzgibbons et al. analyzed the gene-expression profiles of epicardial adipose tissue of patients with coronary artery disease and found no increase in inflammatory gene expression [63]. Whether a link between macrophage polarization in PVAT and plaque composition exists remains unclear and promotes future investigations.

To the best of our knowledge, this is the first study to examine the association between ghrelin expression in the lower-extremity atherosclerotic plaque and PVAT in relationship with plaque components and macrophage polarization state. Ghrelin and its receptor (GHSR) are highly expressed in the heart and vascular system, with immunocytochemical evidence that ghrelin is synthesized by endothelial cells [30].

An earlier study demonstrated that exogenous administration of ghrelin may suppress the expression of anti-inflammatory cytokines in macrophages [64], but the literature is scarce regarding the role that endogenous ghrelin plays in atherosclerosis. Animal studies suggest that GHSR density in the cardiovascular system was 30–40% higher in atherosclerotic coronary arteries compared with normal vessels [65], whereas in the calcified aorta, both expressions of ghrelin and its receptor are decreased [66]. In a mouse model of ghrelin receptor deficiency, atherosclerotic plaques showed increased expression of CD4+ T cells, macrophages, pro-inflammatory molecules, and lower smooth muscle cell expression. These results suggest that GHSR deficiency may aggravate vascular inflammation and plaque instability [40].

The role of the GHSR in mediating the effects of ghrelin in adipose tissue inflammation is still unclear. Previously, a positive association between the immunohistochemical expression of GSHR and CD68-macrophage infiltration was shown in the epicardial adipose tissue of patients with obesity undergoing coronary artery bypass graft surgery [67,68]. In the current study, we noted a greater ghrelin expression in complicated plaques and in the PVAT surrounding complicated plaques, although this finding was not statistically significant. We suggest that this increase may be due to a regulatory anti-inflammatory and anti-apoptotic mechanism [31], since complicated plaques are associated with features of instability and greater pro-inflammatory macrophage content.

The limitations of the study include the small cohort of patients, which may have affected the statistical significance of the results. Co-localization of CD68+ cells with a panel of M1 and M2 macrophage markers would have been useful for a specific and comprehensive analysis of the proportion of macrophage types within the plaque and PVAT. Lastly, ghrelin staining was performed in selected cases (n = 18), and lipid-poor plaques were not evaluated. While current evidence suggests that ghrelin holds a wide array of beneficial antiatherogenic and anti-inflammatory properties, further research is necessary to underly the pathological mechanisms of endogenous ghrelin in advanced atherosclerosis.

## 5. Conclusions

Ghrelin has demonstrated positive effects on vascular function and atherosclerosis development by mediating endothelial dysfunction, inflammation, and oxidation. The relationship between ghrelin and macrophage inflammatory infiltrate in the PVAT of patients with CLI cannot be directly derived from our current findings. Nonetheless, we noted increased ghrelin staining in both complicated plaques with features of instability and PVAT surrounding the respective plaques, suggesting ghrelin’s involvement in inflammation driving advanced atherosclerosis. Additionally, our findings support prior reported involvement of the pro-inflammatory M1 macrophages in plaque instability and higher plaque lipid composition. As this study is the first to relay ghrelin expression in PVAT and plaques in patients with CLI, our data require further investigations to confirm and expand on our results.

## Figures and Tables

**Table 1:** Characteristics of the study participants.

Description of the Study Group	Patients/Years/Gender
Patients	28
Age	69 (53–86 *)
Male	21 (75%)
PAD risk factors	
Hypertension	25 (89.28%; 18 M. 7 F.)
Obesity	5 (17.85%; 3 M. 2 F.)
Diabetes	13 (46.42%; 11 M. 2 F.)
Dyslipidemia	9 (32.14%; 6 M. 3 F.)
Smoking	20, of which 1 former smoker (71.42%; 17 M. 3 F.)
Chronic kidney disease	4 (14.28%; 3 M. 1 F.)
Vascular complications	
Previous arterial surgery	5 (17.85%; 3 M. 2 F.)
Diabetic foot	3 (10.71%; 2 M. 1 F.)
Sepsis and gangrene	5 (17.85%; 3 M. 2 F.)

Abbreviations: F. female, M. male. * Minimum and maximum age.

**Table 2:** Univariate analysis of plaque types according to age group and gender.

	Fibrous Plaque	Fibro-Lipidic Plaque	*p*-Value	Uncomplicated Plaque	Complicated Plaque	*p*-Value
Age			0.666			0.207
50–59 y. o.	2	3		0	5	
60–69 y. o.	5	5		4	6	
70–79 y. o.	5	3		1	7	
≥80 y. o.	4	1		0	5	
Gender			0.184			0.082
F.	6	1		3	4	
M.	10	11		2	19	

Abbreviations: y. o. years old, F. female, M. male.

**Table 3:** Distribution of complication according to plaque type.

Complication	Fibrous Plaque	Fibro-Lipidic Plaque	*p*-Value
Calcification	7	12	0.003
Ulceration	3	7	0.445
Thrombosis	5	7	0.445

**Table 4:** Distribution and inflammation type according to plaque type and its perivascular tissue.

	Plaque		*p*-Value	Plaque		*p*-Value	Periart.		*p*-Value	Periart.		*p*-Value
	f. p.	f-l. p.		u. p.	c. p.		f. p.	f-l. p.		u. p.	c. p.	
**Inflammation**	8 8 0	1 9 2	0.027	5 0 0	4 17 2	0.002	10 6 0	6 4 2	0.216	4 1 0	10 9 2	0.585
0 1 2												
**Inflammation type**	7 1	8 3	0.052	0 0	15 4	0.002	3 3	3 0	0.148	0 0	8 3	0.795
l-p. polymorphous												

Abbreviations: c. p. complicated plaque, f. p. fibrous plaque, f-l. p. fibro-lipidic plaque, l-p. lymphoplasmacytic, Periart. perivascular, u. p. uncomplicated plaque.

**Table 5:** Univariate analysis of CD68, CD80, and ghrelin according to plaque type and its perivascular tissue.

	Plaque		*p*-Value	Plaque		*p*-Value	Periart.		*p*-Value	Periart.		*p*-Value
	f. p.	f-l. p.		u. p.	c. p.		f. p.	f-l. p.		u. p.	c. p.	
**CD68**			0.017			0.074			0.809			0.367
0	8	0		4	4		7	3		3	7	
1	3	4		0	7		3	3		0	6	
2	1	1		0	2		4	2		2	4	
3	4	7		1	10		2	2		0	4	
**CD80**			0.007			0.272			0.594			1.000
0	10	1		4	7		9	7		4	12	
1	4	4		1	7		5	1		1	5	
2	1	6		0	7		1	1		0	2	
3	1	1		0	2		1	1		0	2	
**Ghrelin inten.**			0.097			0.562			0.209			1.000
0	4	1		1	4		5	5		1	9	
1	0	3		0	3		0	3		0	3	
2	2	5		0	7		1	0		0	0	
3	1	0		0	1		0	0		0	1	
**Ghrelin prop.**			0.067			1.000			0.209			1.000
0	4	1		1	4		5	5		1	9	
1	0	5		0	5		0	3		0	3	
2	1	0		0	1		0	0		0	0	
3	2	3		0	5		1	0		0	1	

Abbreviations: c. p. complicated plaque, f. p. fibrous plaque, f-l. p. fibro-lipidic plaque, inten. intensity, prop. proportion, periart. perivascular, u. p. uncomplicated plaque.

**Table 6:** Distribution of ghrelin score in uncomplicated/complicated plaques and their perivascular tissue.

	Plaque		*p*-Value	Periart.		*p*-Value
	u. p.	c. p.		u. p.	c. p.	
**low** **high**	1 0	4 11	0.313	1 0	7 4	1.000

Abbreviations: c. p. complicated plaque, periart. perivascular, u. p. uncomplicated plaque.

## Data Availability

Data are unavailable due to privacy and ethical restrictions.
